# Supplementary figures and images for: Microstructural profiles of the human superficial white matter and their associations to cortical geometry and connectivity
Source: PLoS Biol. 2026 Jan 30;24(1):e3003629. doi: 10.1371/journal.pbio.3003629 (PMC12885375; doi:10.1371/journal.pbio.3003629)

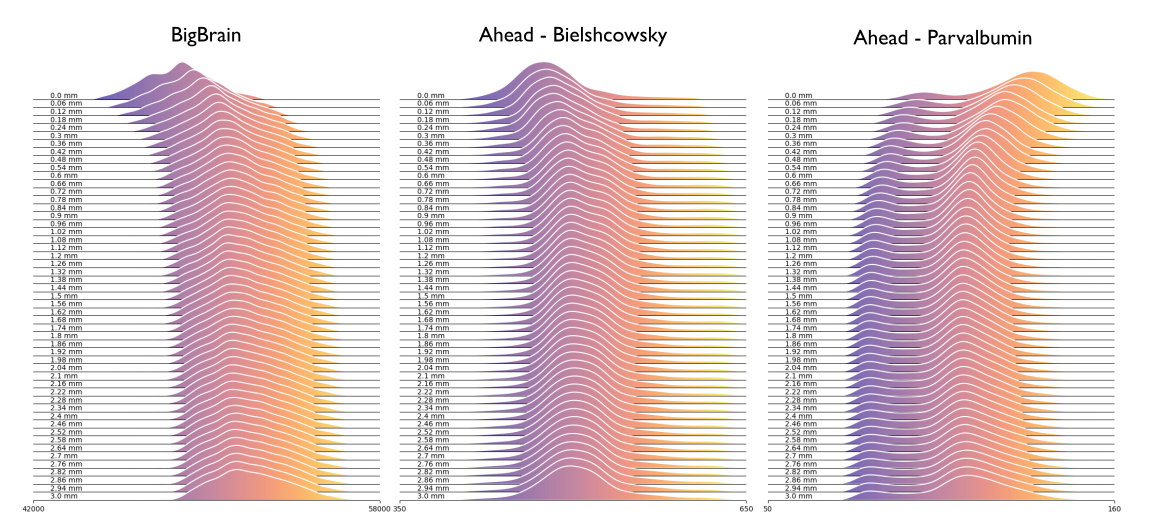

Supplement: S1 Fig — (TIFF) [file pbio.3003629.s001.tiff]

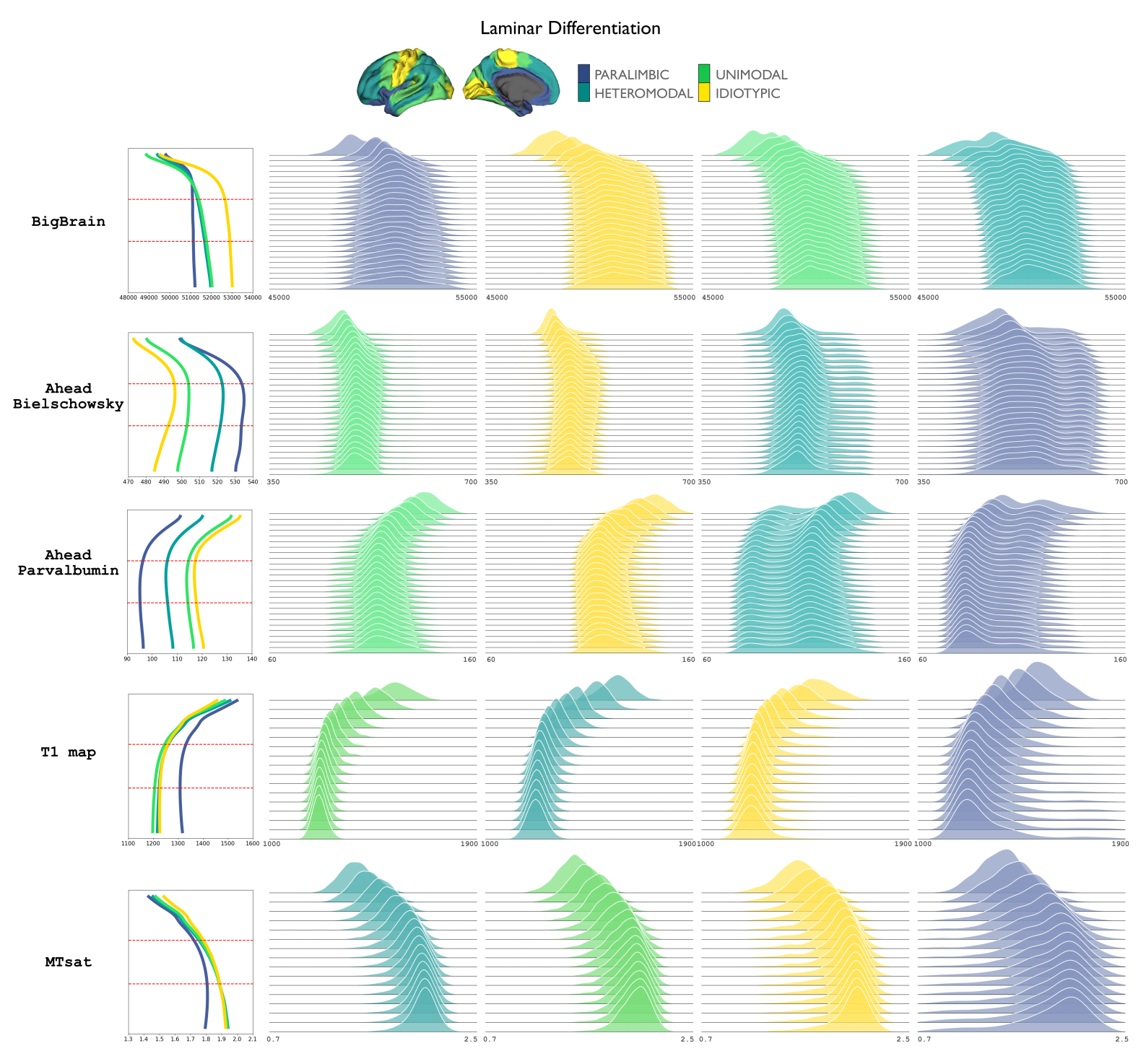

Supplement: S2 Fig — (TIFF) [file pbio.3003629.s002.tiff]

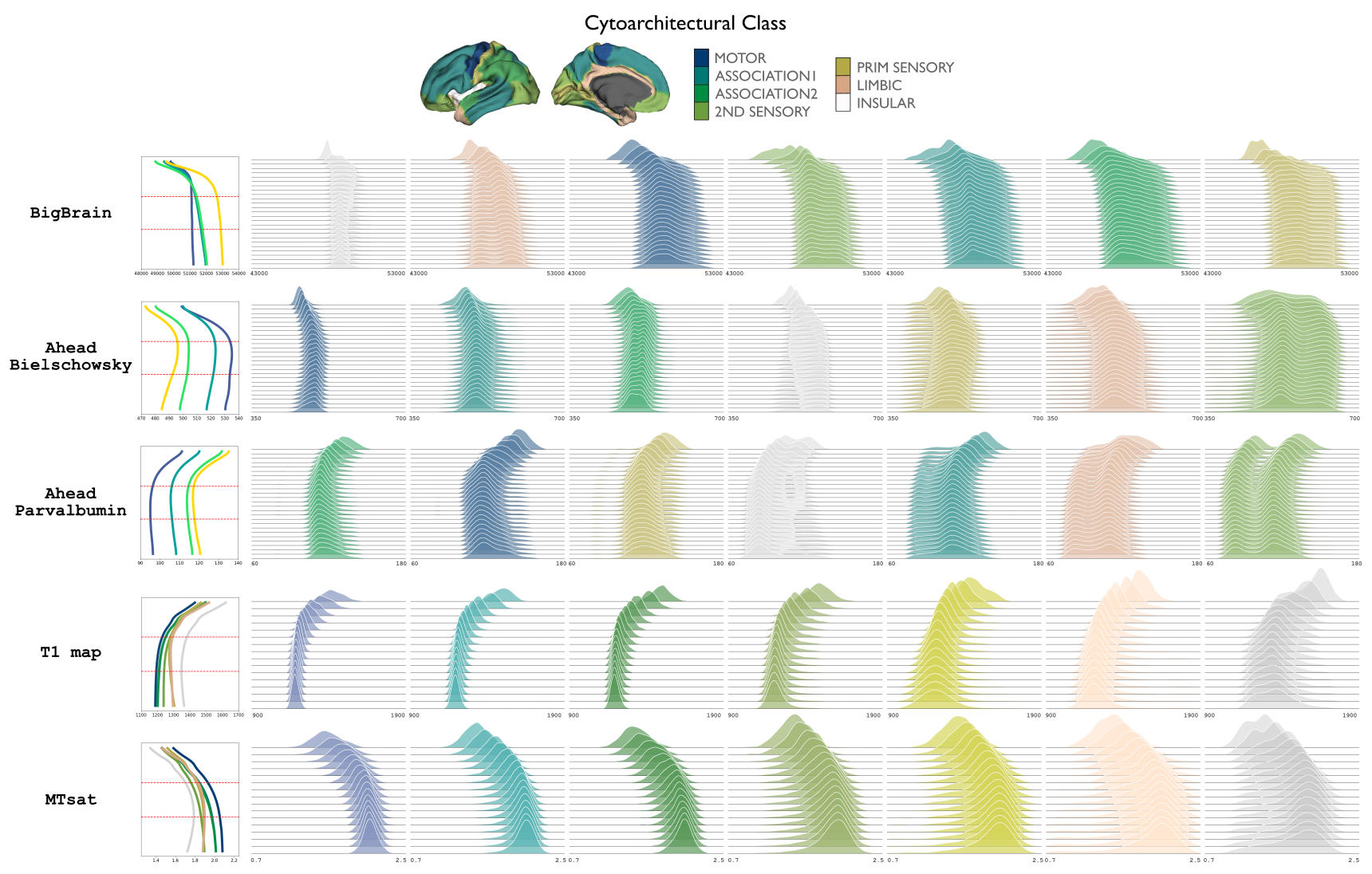

Supplement: S3 Fig — (TIFF) [file pbio.3003629.s003.tiff]

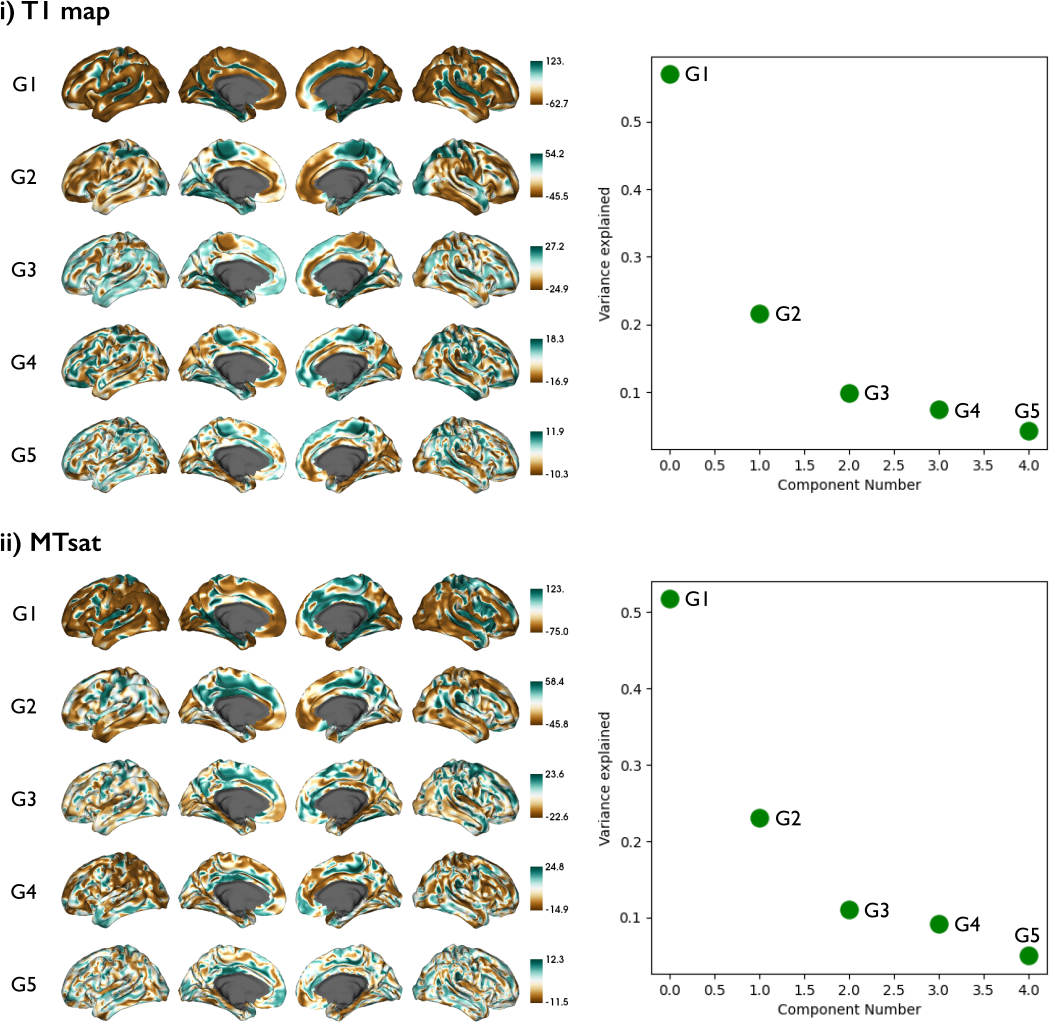

Supplement: S4 Fig — Scree plots show the eigenvalue spectrum for (i) T1 map and (ii) MTsat, illustrating the relative variance explained by each gradient. The data underlying this figure can be found in S1 Data. (TIFF) [file pbio.3003629.s004.tiff]

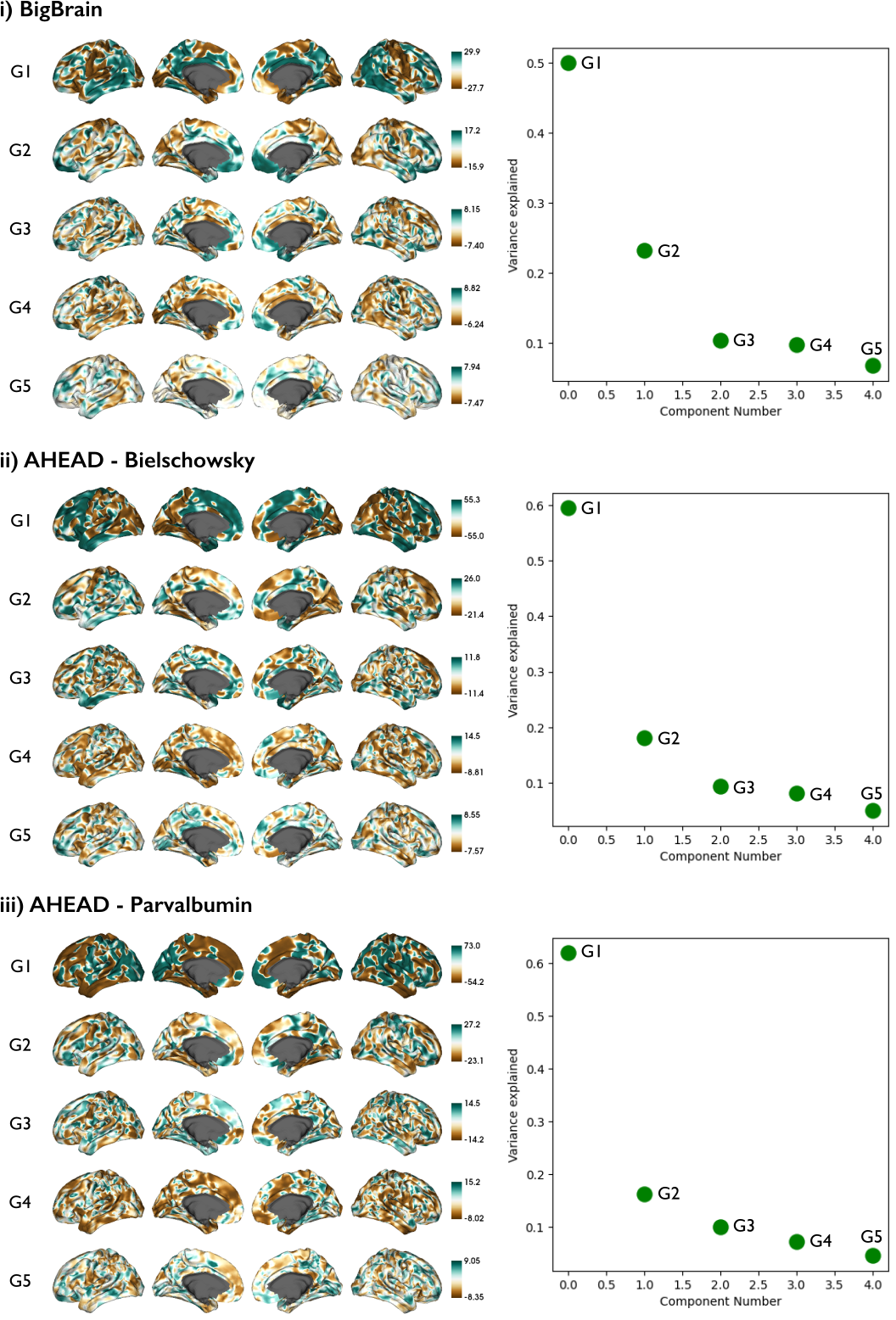

Supplement: S5 Fig — Scree plots display the eigenvalue spectra for (i) BigBrain and (ii) Ahead—Bielschowsky, and (iii) Ahead—Parvalbumin, illustrating the proportion variance explained by each gradient. The data underlying this figure can be found in S1 Data. (TIFF) [file pbio.3003629.s005.tiff]

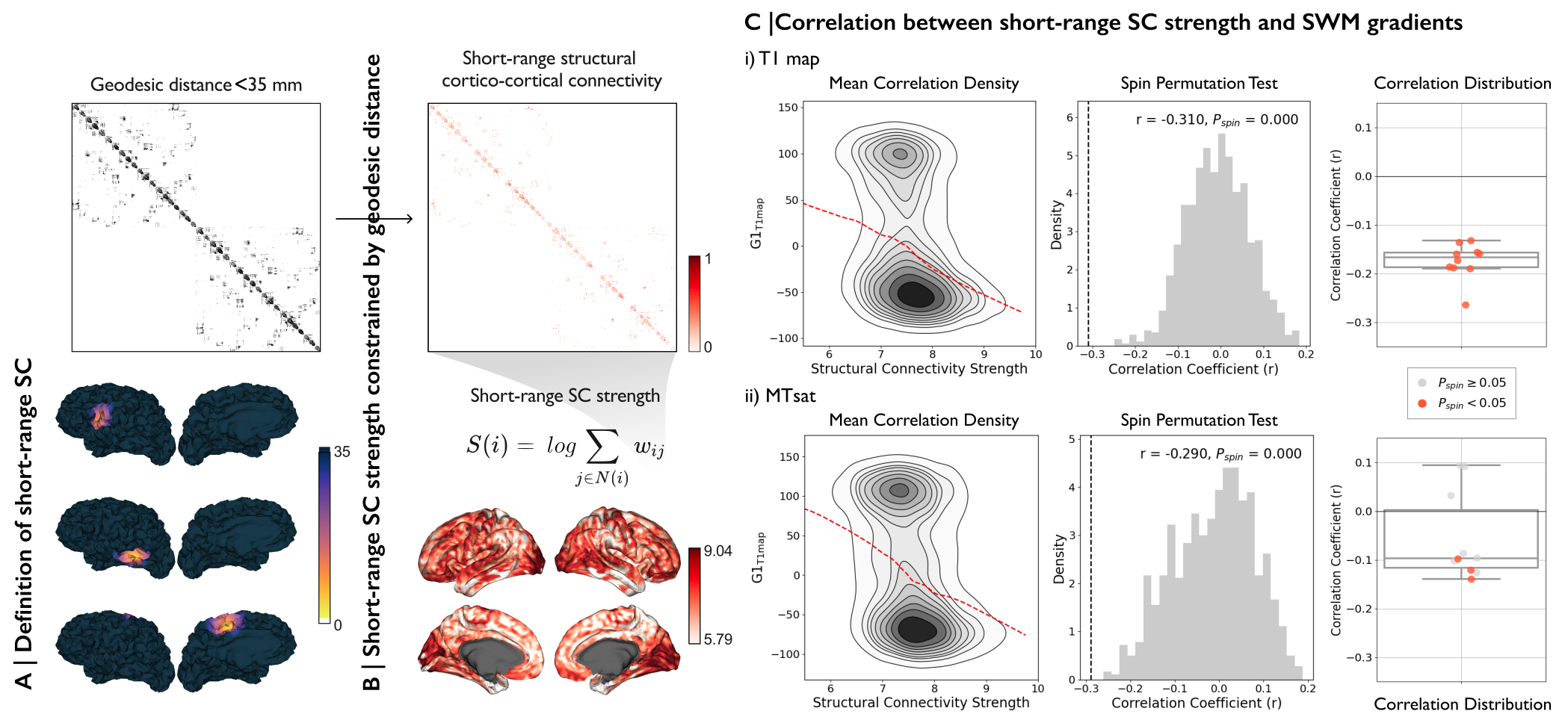

Supplement: S6 Fig — (A) Connections with a geodesic distance <35 mm were selected to capture short fibers (top panel). Examples of three randomly selected vertices with geodesic distances <35 mm are shown in the bottom panel. (B) SC was restricted to this geodesic distance to isolate short-range connections (top panel). Short-range SC strength was calculated as the weighted sum of the short-range SC matrix across all nodes (bottom panel). (C) The left panel shows density plots of nonlinear correlations between connectivity strength and SWM gradients in the group analysis, with statistical significance assessed using spin permutation tests. The red dotted line represents the nonlinear fit capturing the relationship between gradients and connectivity strengths. The right panel presents subject-wise correlations between connectivity strength and SWM gradients derived from each qMRI. Box-and-whisker plots illustrate the distribution of Spearman correlation coefficients across the subjects, with nonsignificant correlations shown as light gray dots. The data underlying this figure can be found in S1 Data. Abbreviations: r, Spearman correlation; Pspin, Significance. (TIFF) [file pbio.3003629.s006.tiff]

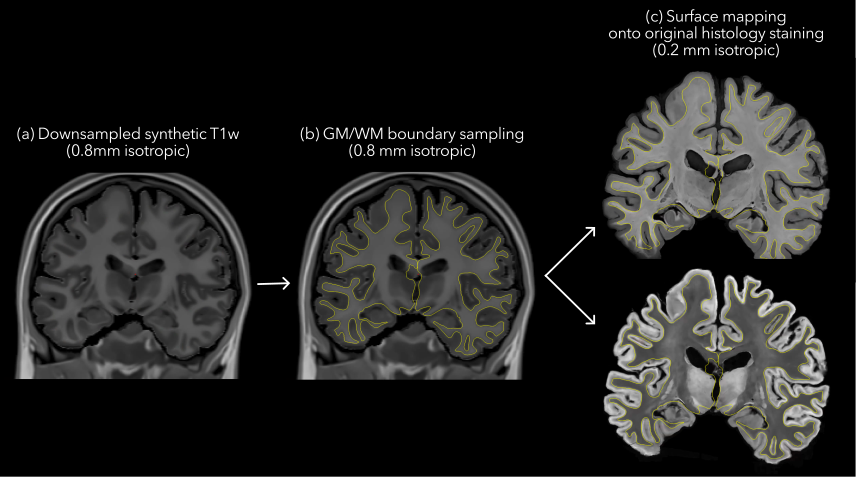

Supplement: S7 Fig — (a) The blockface image was downsampled to 0.8 mm resolution to generate a synthetic T1w image. (b) The GM/WM boundary was delineated on the downsampled blockface image. (c) The GM/WM surface and SWM surfaces were subsequently mapped onto the original histological stainings acquired at 0.2 mm isotropic resolution. (TIFF) [file pbio.3003629.s007.tiff]
