# Supplementary material for: Microstructural profiles of the human superficial white matter and their associations to cortical geometry and connectivity
Source: PLoS Biol. 2026 Jan 30;24(1):e3003629. doi: 10.1371/journal.pbio.3003629 (PMC12885375; doi:10.1371/journal.pbio.3003629)
Supplement: S1 Text — (DOCX) [file pbio.3003629.s009.docx]

**Abbreviations**. **SWM**, superficial white matter; **MRI**, magnetic resonance imaging; **T1 map**, T1 relaxometry; **MTsat**, magnetization transfer saturation; **GM**, gray matter; **WM**, white matter; **CSF**, cerebrospinal fluid; **SD**, standard deviation; **qMRI**, quantitative magnetic resonance imaging; **FC**, functional connectivity; **SC**, structural connectivity; **MPC**, microstructural profile covariance; ***r*,** Spearman correlation; ***P*_spin_**, p-value as determined by a spin test that controls for spatial autocorrelation. **G1**, primary gradient
