# Supplementary material for: Microstructural profiles of the human superficial white matter and their associations to cortical geometry and connectivity
Source: PLoS Biol. 2026 Jan 30;24(1):e3003629. doi: 10.1371/journal.pbio.3003629 (PMC12885375; doi:10.1371/journal.pbio.3003629)
Supplement: Attachment — Submitted filename: response_SWM_PLOSBiology_final.docx [file pbio.3003629.s010.docx]

**Response to Editors and Reviewers (PBIOLOGY-D-25-01584R1)**

We would like to thank the Editors for the opportunity to submit a revised manuscript, as well as the Editors and Reviewers for their thoughtful evaluations of our paper. We are grateful for their constructive comments and found the suggestions very helpful to improve the quality of our paper. We addressed all suggestions in a point-by-point fashion and highlighted the corresponding changes in the manuscript in yellow.

**Reviewer #1**

**The authors introduced a unified framework to study superficial white matter (SWM) organization based on 3D histological and high-field 7T MRI. Using the histology datasets, the authors first characterized depth-dependent variations in cellular staining, which were then analogously studied using in-vivo MRI measures including T1 relaxometry and magnetization transfer saturation. Then, they observed that the SWM principal gradient map derived from T1 exhibited significant spatial correlations with cortical geometry, as well as with structural and functional connectivity strength. The study thus provides novel insights into the organization of SWM in the human brain and underscores the potential of SWM mapping to advance fundamental and applied neuroscience research.**

**The topic is interesting and the open-science approach is commendable. The paper is also well-written. That being said, I have several comments, particularly related to, justifications of but the proposed "unified framework" is confusing due to insufficient justification for many analytical choices.**

We thank the Reviewer for the positive evaluation of our work and for the constructive feedback.

**Major concerns:**

1) **The "unified framework" is a source of confusion as the relationship between the histological and MRI components is not clearly established. The analyses of histological data (Fig. 1) and MRI data (Figs. 2, 4, 5) are presented in parallel, but the manuscript lacks a direct comparison or validation to show how they are integrated. This makes it difficult to understand the contribution of the histology to the final conclusions, which are derived almost exclusively from the MRI data. We suggest the authors revise their description of the framework to more accurately reflect the workflow. For instance, framing it as a "histology-informed" or "conceptually-linked" approach, rather than a "unified" one, would better represent the study's design and make the findings clearer.**
   We agree that the original description as a “unified framework” may have caused confusion regarding the relationship between the histology and MRI components. To address this concern, we changed the *Title* to:

***“A computational framework to study superficial white matter architecture in the human brain on 3D histology and high-field MRI”***

2) **MTsat is generally considered more specific to myelin content than the T1 map, yet it shows weaker correlations with structural connectivity strength (Fig. 5B). How can this be explained? The authors should provide further discussion on this point.**

There could be several reasons for the observed discrepancy. First, the spatial resolution of the T1 map (0.5 mm isotropic) is higher than that of MTsat (0.7 mm isotropic), which may result in more accurate sampling of superficial white matter. Second, the T1 map is sensitive to a broader range of microstructural features beyond myelin, including variations in iron content, which are particularly relevant for superficial white matter architecture. This broader sensitivity may allow T1-based gradients to capture aspects of SWM organization that are not as strongly reflected in MTsat. Finally, since the surfaces used for sampling were derived from the T1-weighted image that was also used to compute the T1 map, the T1-derived surfaces may be inherently more aligned with the T1 map than with MTsat, even though registration accuracy between modalities appeared satisfactory. This point has been added to the *Discussion* (P.13).

*“This effect was more robust for the T1 map than for MTsat. One possible explanation is that the T1 map has higher spatial resolution and is sensitive to a broader range of microstructural features, including iron, which may be particularly relevant for SWM organization.”*

3) **In the Discussion (page 13, paragraph 2), the authors interpreted the correlation between the T1 map gradient and structural connectivity (SC) strength as reflecting the contribution of U-fibers. However, as SC was reconstructed using global tractography, short-range U-fibers are mixed with long-range association and projection fibers. It would be helpful if the authors selected only short streamlines and tested whether the correlations between SC and the T1 map gradient become stronger.**

In line with the Reviewer’s suggestion, we repeated the analyses based on connections with a geodesic distance <35 mm. As shown in the new **Supplementary Figure 6**, short-range SC strength demonstrated a correlation with G1_T1map_ in the group-level analyses (*r* = -0.31, *P_spin_* < 0.05). At the subject level, this relationship remained consistent (mean *r* = -0.17 across subjects, *P_spin_* < 0.05 for all subjects). G1_MTsat_ also showed a correlation with short-range SC strength at the group level (*r* = -0.29, *P_spin_* < 0.05), but this effect was not consistent (mean *r* = -0.05, *P_spin_* < 0.05 in 3 subjects only). Overall, results indicate that the T1 map gradient retains a broadly similar relationship with short-range SC as observed in the original whole-connectome analysis.

**Supplementary Fig. 6. Short-range structural connectivity (SC) correlations with SWM gradients. (A)** Connections with a geodesic distance <35mm were selected to capture short fibers (*top* panel). Examples of three randomly selected vertices with geodesic distances <35 mm are shown in the *bottom* panel. **(B)** SC was restricted to this geodesic distance to isolate short-range connections (*top* panel). Short-range SC strength was calculated as the weighted sum of the short-range SC matrix across all nodes (*bottom* panel). **(C)** The *left* panel shows density plots of non-linear correlations between connectivity strength and SWM gradients in the group analysis, with statistical significance assessed using spin permutation tests. The red dotted line represents the non-linear fit capturing the relationship between gradients and connectivity strengths. The *right* panel presents subject-wise correlations between connectivity strength and SWM gradients derived from each qMRI. Box-and-whisker plots illustrate the distribution of Spearman correlation coefficients across then subjects, with non-significant correlations shown as light gray dots. **Abbreviations**: *r*, Spearman correlation; *P*_spin_, Significance.

Please also see the corresponding revised *Result*s (P.10):

*“Overall, G1_T1map_ demonstrated consistent and significant correlations across both group- and subject-level (see* ***Supplementary Fig. 6*** *for the preliminary short-range structural connectivity analysis), while G1_MTsat_ results showed weaker and less consistent correlation.”*

and the revised *Discussio*n, where we now also outline the need for additional follow-up work to interrogate the U-fiber system (P.14):

*“While this study focused on characterizing surface-based SWM properties, analyzing short-range structural connectivity indicated that the SWM properties retain their relationship with local connectivity patterns. Building on this, future research will incorporate tractography-based approaches to directly investigate the organization and functional role of short-range, U-fiber bundles in SWM connectivity.”*

4) **In Section 4, "Generation of SWM microstructural gradients," the rationale for using a diffusion map embedding approach should be clarified. Could the authors briefly explain what advantages this gradient-based analysis offers over more direct methods, such as analyzing SWM intensity values at specific depths? This would help readers better understand the motivation behind this key analytical step.**

We thank the Reviewer for this opportunity to clarify our rationale. While depth-specific intensity profiles provide valuable information, they capture microstructural variations only at discrete sampling points. In contrast, the gradient-based analyses can map out spatial axes of depth-specific changes in a fully data driven manner.

Please see the revised *Results* (P.9):

*“As spatial patterns of SWM microstructure were revealed, we examined their relationship to cortical geometry. A non-linear dimensionality reduction approach provides a low-dimensional representation of spatial organization, allowing us to capture continuous axes of variation that may reflect fundamental principles of SWM architecture. Using microstructural gradients, we derived gradients that capture spatial pattern of SWM microstructural similarity (****Fig. 4A****).”*

*Discussion* (P.12):

*“While depth-specific intensity profiles provide valuable information, they capture microstructural variations only at discrete sampling points. To overcome this limitation, we applied a diffusion map embedding approach, which allows identification of continuous gradients of microstructural similarity across the SWM depths.”*

and *Methods* (P.18):

*“This approach revealed eigenvectors (or “gradients”) that represent the primary spatial axes of variance in SWM microstructural profile similarities^70,71^. Such gradients provide a low-dimensional representation of SWM organization, highlighting macroscale patterns of microstructural variation.”*

**Minor Concerns:**

5) **Regarding the density maps in Figure 5B (middle panel), the visualization could be clarified. The plot shows high data density for structural connectivity strengths greater than 6, but the red trend line extends down to values around 4, where data appears to be sparse or absent. Please adjust the line to better reflect the range of the underlying data or clarify this in the caption.**

As suggested, we updated **Figure 5B** to ensure that the red trend line is restricted to the range where sufficient data points are available.

**Fig. 5. Functional connectivity (FC) and structural connectivity (SC) correlation analysis with SWM gradients. (A)** FC and **(B)** SC strength were calculated as the weighted sum of the connectivity matrix across all nodes (*left* panel). Matrices were sorted by regions with greater similarity and nonlinearity. The *middle* panel shows the density plot of non-linear correlations between connectivity strength and SWM gradients in the group analysis, along with statistical significance assessed using a spin permutation test. The red dotted line in the density plots represents a non-linear fit capturing the relationship between the gradients and the connectivity strengths. The *right* panel presents subject-wise correlations between each connectivity strength and the SWM gradients from each MRI. Box-and-whisker plots illustrate the distribution of Spearman correlation coefficients across ten subjects, with non-significant correlations shown as light gray dots. **Abbreviations**: *r*, Spearman correlation; *P*_spin_, Significance.

6) **Please report the variance explained by the principal gradients. A scree plot showing the eigenvalues for the leading gradients would be helpful to justify the focus on G1.**

We now include a scree plot (**Supplementary Figure 4**) displaying the eigenvalue spectrum.

**Supplementary Fig. 4. Variance explained by gradients of microstructural profile covariance from the *in-vivo* MRI datasets.** Scree plots show the eigenvalue spectrum for *(i)* T1 map and *(ii)* MTsat, illustrating the relative variance explained by each gradient.

And refer to these findings in the *Results* (P.9)

*“The primary gradient (G1) explained 57% of the variance in the T1 map and 52% in MTsat, highlighting its dominant contribution relative to higher-order gradients (****Supplementary Fig. 4****).”*

7) **Please clarify the rationale for correlating SWM gradients with connectivity node strength. The principal gradient represents a global axis of microstructural organization, whereas node strength is a local, summary metric of connectivity. What is the underlying hypothesis that connects these two different scales of brain organization?**

We thank the Reviewer for this insightful comment. While the principal gradient of SWM microstructure reflects a global axis of microstructural organization, node strength provides a summary metric of each region’s connectivity, capturing the cumulative influence of local and long-range connections. In our study, connectivity strength was computed as the weighted sum of the connectivity matrix across all nodes, which inherently incorporates information from the entire network. Therefore, although it is a regional metric, it reflects a region’s integration within the global connectivity structure. Our underlying hypothesis was that variations along the gradient potentially exhibiting distinct roles in both local and large-scale network communication. This approach allows us to probe the interplay between microstructural organization and macroscale connectivity patterns.

Please also see the revised *Discussion* (P.13):

*“Our findings also demonstrated a correlation between the T1 map gradient and cortico-cortical functional and structural connectivity in the SWM region. To bridge microstructural and macroscale perspectives, we examined how the principal gradient of SWM microstructure relates to regional connectivity strength. As this measure reflects each region’s integration within the global connectome, our underlying hypothesis was that variations along the gradient may correspond to distinct roles in both local and large-scale network communication. This approach allows us to probe the interplay between microstructural organization and macroscale connectivity patterns.”*

8) **There appears to be a noteworthy contrast in the findings for MTsat and structural connectivity (Figure 5Bii). While the group-level correlation is reported as significant (P_spin = 0.024), the plot of individual correlations suggests the effect is non-significant in most subjects. Could the authors please comment on this? Clarifying whether this is due to a consistent but weak effect across subjects would help in the interpretation of the overall result.**

Following the comment, we reprocessed the MTsat data using a minimal-dependency Python pipeline. This resulted in more consistent and also associated to stronger subject-level correlations, with a greater number of participants showing significant effects. Nevertheless, some subjects remained non-significant. We believe this discrepancy arises because the group-level correlation reflects a consistent effect across subjects that becomes statistically robust when aggregated, whereas individual subject-level analyses are more susceptible to noise and inter-individual variability, resulting in some non-significant findings.

Please also see the revised *Results* (P.10):

***“****By contrast, G1_MTsat_ showed a correlation with structural strength in the group analysis (r = -0.312, P_spin_ = 0.024), and most individual subjects did not show significant correlations (mean r = -0.025 across subjects, P_spin_ < 0.05 for 6 subjects).****”***

**Reviewer #2**

**The paper entitled "A unified imaging-histology framework for superficial white matter architecture studies in the human brain" is among the few studies that have tackled the superficial white matter (SWM) and a first effort to systematically study and compare SWM architecture across the entire cortex at multiple scales, using a multimodal approach that combined high resolution microscopy and histology with several high-field MRI datasets and in vivo scans. The authors analyzed both intensity profiles and statistical moments and the findings showed high structural complexity that varied across architectural gradients and showed variable correlations with structural and functional connectivity.**

**This is a very timely, interesting, and well written study and I want to commend the authors on their effort, care, and consideration of relevant literature, while completing this much-needed work. The strengths of this manuscript include (1) the impressive integration of two high-resolution histological microscopy datasets with several high-field 7T MRI and other in vivo structural, diffusion-weighted and functional MRI sets, (2) the rigorous approach used for SWM surface-based sampling that utilized the Laplacian vector field within the white matter domain, as well as approaches used to link systematic variations with cortical geometry and connectivity, and (3) the development of a very useful open access framework that can be widely used in future studies.**

**The findings highlight the intricate nature of SWM, are novel, and highly significant for understanding the relationship between architecture/structure, connectivity patterns, and function. They are a very important contribution not only to basic neuroscience but also for the future study of atypical function and pathology, and as such, the paper is of interest to both basic and clinical researchers. Importantly, the study's findings provide a foundation for future comparative studies, normative neuroimaging studies, and studies of white matter pathology in disorders.**

**Below few comments and suggestions that in my opinion will further increase the value and clarity of the manuscript, improve presentation, and strengthen reported findings and discussion:**

We thank the Reviewer #2 for the positive assessment of the paper.

**Results and Methodological considerations**

1) **50 fields were analyzed from the histological datasets; however, fewer were shown in Fig. 1B. Why not all? Please clarify and justify selection.**

We apologize for the confusion. Displaying all 50 ridge plots would make the figure overly large and a bit more difficult to interpret. Therefore, we selected every 0.12 mm (resulting in 25 SWM surfaces) to illustrate intensity variations. To nevertheless ensure completeness, we now provide the full set of 50 fields in **Supplementary Figure 1**.

**Supplementary Fig. 1.** Alterations in intensity distributions of histological features across SWM depths, from the GM/WM interface to 3 mm into the SWM.

We also revised the caption of **Figure 1B** to clarify this point (P.4):

*“****Fig. 1.*** *…* ***(B)*** *Alterations in the intensity distributions of histological features across SWM depths. The GM/WM interface (0 mm) and 25 representative SWM surfaces (up to 3 mm into the white matter) are shown. The full set of 50 SWM surfaces, and their intensity alterations are provided in* ***Supplementary Fig. 1****. The surfaces showing the greatest variation in SWM, from 0 mm to 1.8 mm into the white matter, were mapped onto the brain alongside the intensity profiles on the left. …”*

2) **Similarly, 15 surfaces were analyzed from the MRI datasets however, Fig. 2B shows data from 16 surfaces. Which is it?**

Fifteen SWM surfaces were sampled from the *in-vivo* MRI dataset. The first surface shown in Figures 1B and 2B corresponds to the GM/WM interface. We revised the caption of **Figure 2B** to clarify this point and avoid further confusion (P.6):

*“****Fig. 2.*** *…* ***(B)*** *Alterations in the intensity distributions of MRI features across SWM depths. The GM/WM interface (0 mm) and 15 SWM surfaces (up to 3 mm into the white matter) are shown. The surfaces showing the greatest variation in SWM, from 0 mm to 2 mm into the white matter, were mapped onto the brain alongside the intensity profiles on the left. …”*

3) **Results, Section 3, page 7: The authors state "The consistently low SD observed at the GM/WM interface across most of modalities likely reflects a shared anatomical transition across cortical areas, where similar intensity changes yield reduced regional variability". Which modalities did not show consistently low SD?**

Thank you for noting this. Specifically, MTsat showed a trend toward increased SD at the GM/WM interface, unlike the other modalities. We have clarified this point in the *Results* (P.7):

*“The consistently low SD observed at the GM/WM interface across most modalities, with the exception of MTsat, likely reflects a shared anatomical transition across cortical areas, where similar intensity changes yield reduced regional variability.”*

4) **Results, Sections 4 and 5, pages 9 and 10 (associations and correlations): Since both MRI and histological datasets were eventually aligned to standard templates, why were these analyses only conducted on the MRI and not the histological datasets?**

Our main rationale was that MRI data allow us to extract morphological information and extend analyses to functional and structural connectivity. While histological gradients could also be computed, the histology and MRI datasets come from different subjects, making direct integration in the main analyses less appropriate. Nevertheless, to address this point, we now provide histology-based gradient analyses in **Supplementary Figure 5** for reference.

**Supplementary Fig. 5. Variance explained by gradients of microstructural profile covariance from the histology datasets.** Scree plots display the eigenvalue spectra for *(i)* BigBrain and *(ii)* Ahead – Bielschowsky, and *(iii)* Ahead – Parvalbumin, illustrating the proportion variance explained by each gradient.

We clarified this point in *Results* to make it explicit (P.9)

*“For reference, we also computed histology-based gradients (****Supplementary Fig. 5****)”*

5) **Materials and Methods, Section 1b, page 14: Since images were heavily preprocessed and downsampled, it would be useful to show example images in supplementary materials.**

The images were preprocessed and downsampled to enable surface sampling at the CSF/GM and GM/WM boundaries. However, the surfaces themselves were mapped from the original high-resolution histology (0.2 mm for Ahead dataset). To provide clarify, we now include representative preprocessed and native-resolution images in **Supplementary Figure 7**.

**Supplementary Fig. 7.** (a) The blockface image was downsampled to 0.8 mm resolution to generate a synthetic T1w image. (b) The GM/WM boundary was delineated on the downsampled blockface image. (c) The GM/WM surface and SWM surfaces were subsequently mapped onto the original histological stainings acquired at 0.2 mm isotropic resolution.

And also updated the *Methods* (P.15):

*“… FastSurfer recommends a T1-weighted (T1w) image as input and has resolution limitations, particularly for histological datasets exceeding a 0.7 mm isovoxel resolution^38^. Accordingly, the blockface image was downsampled to a 0.8 mm isovoxel resolution to generate a synthetic T1w image. This was achieved by inverting the downsampled blockface image, supplementing it with a synthetic head, and processing it to remove cerebrospinal fluid, which could interfere with cortical surface extraction. Additionally, non-local means denoising was applied to enhance contrast prior to running FastSurfer. As a result, we obtained classified volumes as well as CSF/GM and GM/WM surfaces. Subsequently, the original high-resolution images were used to map microstructural properties onto the SWM surfaces (****Supplementary Fig. 7****). …”*

6) **Materials and Methods, Section 1c, page 15: Clarifications and more details needed to describe the approach here for a more general readership. The authors state that the Laplacian field provided an isomorphic mapping between points on the SWM surface and the overlying cortex, enabling seamless integration of gray matter and white matter metrics. Is this how the surface-based sampling was eventually associated with different cortical regions? In other words, how did the authors extend the borders of grey matter regions to the SWM to define limbic, paralimbic, motor, etc. areas? This last comment also applies to the following Methods, section 3. Neural contextualization with cytoarchitecture, which lists Mesulam and vonEconomo cytoarchitectonic maps but the integration of these with the data is not clear.**

To clarify this point, the Laplacian field enabled an isomorphic mapping between the cortex and the underlying SWM. Cortical parcellations (Mesulam and von Economo) were then extended along the surface vertices into the SWM, allowing us to examine how microstructural intensity varies across cortical region types. Because the SWM lies directly beneath the cortical sheet, this approach facilitates the investigation of regional differences relative to established cortical parcellations.

Please see the revised *Methods* (P.18):

*“The SWM intensity profiles derived from histology and MRI were contextualized within the framework of normative variations in cortical cytoarchitecture, which detail the cellular composition and structural characteristics of cortical regions. These intensity profiles were categorized based on cortical types defined by Von Economo and Koskinas^66,67^. We also utilized the atlas proposed by Mesulam^68^ to delineate four functional regions, following variations in cortical laminar differentiation. To map SWM to cortical types, the Laplacian field provided an isomorphic mapping between the cortex and underlying SWM, extending cortical labels along the surface vertices into the SWM.”*

7) **Materials and Methods, Section 1c, page 15: in the same section, the authors state "The maximum SWM depth of 3 mm was chosen to capture both the U-fiber system and the termination zones of long-range bundles". Several sulcal areas in the cortex contain quite narrow white matter regions, such that the SWM is not deep (less than 3mm), often quickly changes to deep white matter and then SWM of a neighboring area/region. Similarly, subgenual cingulate areas (like posterior BA25), have very narrow SWM. Conversely, other cortical areas have a much thicker (deeper) SWM component. How was this tackled and taken this into account for the analysis and interpretation of the findings?**

We selected 3 mm based on prior work demonstrating that this depth captures both U-fibers and the termination zones of long-range fiber bundles (Schüz et al., 2002). We acknowledge that SWM depth varies regionally, however, our aim was to examine depth-dependent intensity variation rather than absolute thickness. Importantly, most regions showed pronounced structural complexity within the first ~ 2 mm, supporting our focus on this depth range. We have clarified this rationale in the revised *Discussion* (P.13)

*“… This indicated that the first 2 mm of the SWM region is characterized by high structural complexity, likely reflecting the interplay between tangentially oriented U-fibers, which run parallel to the GM/WM interface, and radially aligned cortical afferents and efferents, which intersect perpendicularly^41,88^. Beyond approximately 2 mm into the white matter, in most regions, structural complexity appeared to stabilize, consistent with a transition to more homogeneous long fiber bundles characteristic of the deep white matter. …”*

**General and Discussion**

8) **In the Results, Section 3, bottom of page 7, the authors state "Together, these findings indicate that SWM is not a homogeneous region but a structured continuum, exhibiting depth-dependent microstructural variations shaped by cellular, fiber, and myelin organization, which vary across the brain". This finding is later discussed and there is a brief comparison with relevant data from other species. However, it should also be noted that this was independently shown for several WM regions across primates, based on density and distribution of axons and neurons, including cortical WM in chimpanzees (Swiegers et al., J. Comp. Neurol. 2021), cortical WM in rhesus macaques (Mortazavi et al., Frontiers in Neuroanatomy 2017 and2016), PFC WM in humans and non-human primates (Zikopoulos et al., PLOS Biology 2018).**

We now cite the suggested studies in the revised *Discussion* (P.13):

*“Our findings support the notion that the SWM forms a structured continuum rather that a homogeneous region, with depth-dependent microstructural variations shaped by cellular, fiber, and myelin organization across different brain areas, supporting these organizational principles. Similar depth-dependent patterns have also been observed across white matter regions in non-human primates^95,98,99,100^.”*

9) **In the Discussion, page 12, the authors state "Historically, the structural complexity of the SWM has challenged its neural characterization, with its cellular heterogeneity and diverse cytoarchitectonic features varying significantly across regions". The meaning of this sentence is not clear. Structural complexity, cellular heterogeneity and diverse cytoarchitectonic features vary significantly across gray and white matter areas. Complexity is a lot more pronounced in gray matter. Several studies have shown that systematic variability patterns in both gray and white matter can facilitate characterization of brain areas (e.g., classical vonEconomo or Sanides or Flechsig studies, and more recently Huntenburg et al., Cerebral Cortex 2017; Zikopoulos et al., PLOS Biology 2018).**

We appreciate this valuable clarification. Our intent was not to suggest that SWM is more complex than gray matter, but rather that variability in SWM composition (*e.g.,* interstitial neurons, U-fibers, and myelination) contributes to its regional heterogeneity. We revised the sentence to better reflect this nuance and added references to classical and modern studies to highlight that systematic variability across both gray and white matter can indeed facilitate characterization of brain areas (P.12).

*“Historically, the structural complexity of the SWM has challenged its neural characterization^13,75^. However, systematic patterns of variability across both gray and white matter regions have been shown to facilitate the reliable characterization of cortical and subcortical areas^66,94,95^.”*

10) **In the Discussion, page 12, the authors state "These white matter interstitial neurons play a critical role during cortical development and have been reported to be associated with psychiatric disorders, where alterations in their density and distribution have been reported". A major disorder where axon and cell density and patterning are affected is autism (see Avino and Hutsler Brain Research 2010 and 2021).**

We thank the Reviewer for this important clarification. In line with this suggestion, we revised the relevant sentence in the *Discussion* to explicitly mention autism and to cite *Avino and Hutsler, (2010; 2021) Brain Research* (P.12-13):

*“These white matter interstitial neurons play a critical role during cortical development^79,80^ and have been implicated in psychiatric disorders^81,82,83,84^, with autism in particular showing alterations in their density and distribution^96,97^”*

11) **In the Discussion, page 13, the authors state "Specifically, it showed a stronger correlation with structural connectivity, which may reflect the contribution of U-fibers, known to support local cortical communication and as a predominant feature of the SWM". This is in line with data showing major contribution of short-range connectivity in the WM, (see Rosen and Halgren PLOS Biology 2022).**

In the revised manuscript, we now cite *Rosen and Halgren, (2022) PLOS Biology* to support the importance of short-range connectivity as a major contributor to white matter organization, consistent with our interpretation (P.13):

*“Specifically, the T1 map showed a stronger correlation with structural connectivity, which may reflect the contribution of U-fibers, known to support local cortical communication and as a predominant feature of the SWM^101^.”*

**Reviewer #3**

**This work provides a novel characterization of the superficial white matter using both histology and in-vivo MRI data. A surface-based approach was taken to derive the features for characterizing the depth-dependent variations of SWM properties. While this is very valuable work in improving our understanding SWM microstructures, the work mostly provide qualitative and global descriptions and lacks insight on SWM structure. More detailed comments are as follows:**

We thank Reviewer #3 for insightful viewpoints that have helped us refine and solidify our study.

1) **The author claims a "framework" for studying SWM, but it is not clear what is the framework and how this framework is critical for studying SWM.**

In our manuscript, the term “framework” refers to a Python-based, open-source, and reproducible surface-based pipeline and analysis approach that we developed to study SWM. Specifically, the framework enables systematic sampling of SWM surfaces and microstructural covariance with minimal dependencies. Importantly, this approach can be applied consistently to both 3D histology and high-field MRI datasets, ensuring reproducibility and comparability.

We added clarifications to the revised *Introduction* (P.3).

*“In this study, we aimed to develop a framework for densely mapping the microstructural organization of the SWM. Specifically, we designed an open-source surface-based pipeline in Python that enables the systematic sampling of SWM surfaces and microstructural characteristics on both 3D histology and high-field MRI datasets. First, we analyzed the microstructural complexity of the SMW using post-mortem 3D histology to identify key features of cellular architecture and validate our surface-based sampling methodology. …”*

And the revised *Discussio*n (P.12):

*“In this study, we investigated the microstructural integrity of the SWM using 3D post-mortem histology and in-vivo 7T MRI, establishing an openly available framework for SWM exploration. This framework provides a surface-based, reproducible method for sampling SWM intensity profiles and microstructural covariance, while requiring minimal dependencies to ensure wide accessibility. By analyzing intensity profiles and statistical moments across different SWM depths, we identified pronounced structural complexity within the first 2 mm of the SWM, reflecting significant variations in histological staining and myelin-sensitive MRI intensities. …”*

2) **It's valuable to use the surface representation to study the SWM, but only global distributions were provided without providing much details of the local and regional variations of SWM.**

We expanded our analyses to address local and regional variations of SWM microstructure. Specifically, we extended the examination of cellular intensity profiles across SWM depths for both histology and MRI datasets. The results are not further detailed in **Supplementary Figure 2** (Laminar Differentiation) and **Supplementary Figure 3** (Cytoarchitectural class), where SWM intensity distributions are presented across different cortical types. These supplementary analyses reveal depth-dependent differences according to cortical type, allowing us to characterize regional variation in SWM microstructure relative to established cortical classifications. This approach leverages the close anatomical proximity of SWM to the cortical sheet and provides a more fine-grained contextualization of the observed variability.

**Supplementary Fig. 2.** Spatial associations between alterations in intensity distributions and levels of laminar differentiation in histology and MRI features across SWM depths.

**Supplementary Fig. 3.** Spatial associations between alterations in intensity distributions and cytoarchitectural classes in histological and MRI features across SWM depths.

In the *Results* (P.4,6):

*“****Fig. 1 and 2. … (C)*** *Spatial associations between intensity profiles and levels of laminar differentiation (left) and cytoarchitectural class (right). See also* ***Supplementary Figs. 2 and 3*** *for extended analyses of intensity distribution alterations across cortical types.”*

3) **Could the intensity profiles transitioning from GM to WM in Figure 1 and 2 be due to partial volume effects?**

We agree that partial volume effects may contribute, particularly at the GM/WM boundary where the voxel size is larger than our sampling step. However, the GM/WM boundary is relatively less susceptible to this issue than the CSF/GM surface and selected it as a reliable anchor point for depth-dependent profiling.

Importantly, when extending profiles from the CSF/GM surface into the SWM (Figure 3), the observed intensity variations show gradual transitions rather than random blurring across boundaries. This spatially coherent pattern strongly suggests that the profiles capture underlying microstructural differences rather than being solely driven by partial volume effects.

We clarified this point in the revised *Discussion* (P.12):

*“… By analyzing intensity profiles and statistical moments across different SWM depths, we identified pronounced structural complexity within the first 2 mm of the SWM, reflecting significant variations in histological staining and myelin-sensitive MRI intensities. This suggests that the gradual changes seen across depths primarily reflect genuine microstructural variation rather than imaging artifacts and are consistent with significant variations in histological staining and myelin-sensitive MRI intensities. …”*

4) **There is not much synergy in the results from histology and in-vivo MRI except both use the same surface and depth representation to extract SWM related features. Can the same gradient maps be computed for the histology features?**

We agree that direct synergy between histology- and MRI-derived results is limited, primarily because the histology and qMRI datasets originate from different individuals and features. Nevertheless, it is indeed possible to compute gradients from histological features, and we provide these as reference in **Supplementary Fig. 5**. While these histology-based gradients were not integrated with the main analyses, they illustrate the methodological feasibility of extracting microstructural gradients from *post-mortem* 3D histology data and complement our MRI-based group-level analyses, which were necessary for linking SWM microstructure to cortical geometry, structural and functional connectivity.

**Supplementary Fig. 5. Variance explained by gradients of microstructural profile covariance from the histology datasets.** Scree plots display the eigenvalue spectra for *(i)* BigBrain and *(ii)* Ahead – Bielschowsky, and *(iii)* Ahead – Parvalbumin, illustrating the proportion variance explained by each gradient.

We clarified this point in the revised *Results* (P.9):

*“For reference, we also computed histology-based gradients, which are provided in* ***Supplementary Fig. 5****.”*

5) **There is no insight on the principal gradient of the T1 map in Figure 4. Why does it depend on the sulcal/gyral regions? Is the sulcal/gyral separation quantitative or just qualitative observation? The association with cortical geometry is rather convenient but not fundamental.**

For clarity, we revised the *Results* as follows (P.9):

*“Visual inspection of these spatial patterns suggests that the primary gradient derived from the T1 map varies along the main sulci and gyri, while the gradient derived from MTsat appears anchored at one end by the central sulcus, the insula, and the frontal blade^74^* ***(Fig. 4A, right)****.”*

6) **Correlation with functional and structural connectivity is only based on global measures. There is a lack of more specific regional analyses. For structural connectivity, there is a lack of investigation of the impact of tractography techniques given the challenges in obtaining high quality U-fibers in SWM.**

In line with the Reviewer’s suggestion, we repeated the analyses based on connections with a geodesic distance <35 mm. As shown in the new **Supplementary Figure 6**, short-range SC strength demonstrated a correlation with G1_T1map_ in the group-level analyses (*r* = -0.31, *P_spin_* < 0.05). At the subject level, this relationship remained consistent (mean *r* = -0.17 across subjects, *P_spin_* < 0.05 for all subjects). G1_MTsat_ also showed a correlation with short-range SC strength at the group level (*r* = -0.29, *P_spin_* < 0.05), but this effect was not consistent (mean *r* = -0.05, *P_spin_* < 0.05 in 3 subjects only). Overall, results indicate that the T1 map gradient retains a broadly similar relationship with short-range SC as observed in the original whole-connectome analysis.

**Supplementary Fig. 6. Short-range structural connectivity (SC) correlations with SWM gradients. (A)** Connections with a geodesic distance <35mm were selected to capture short fibers (*top* panel). Examples of three randomly selected vertices with geodesic distances <35 mm are shown in the *bottom* panel. **(B)** SC was restricted to this geodesic distance to isolate short-range connections (*top* panel). Short-range SC strength was calculated as the weighted sum of the short-range SC matrix across all nodes (*bottom* panel). **(C)** The *left* panel shows density plots of non-linear correlations between connectivity strength and SWM gradients in the group analysis, with statistical significance assessed using spin permutation tests. The red dotted line represents the non-linear fit capturing the relationship between gradients and connectivity strengths. The *right* panel presents subject-wise correlations between connectivity strength and SWM gradients derived from each qMRI. Box-and-whisker plots illustrate the distribution of Spearman correlation coefficients across then subjects, with non-significant correlations shown as light gray dots. **Abbreviations**: *r*, Spearman correlation; *P*_spin_, Significance.

Please also see the corresponding revised *Result*s (P.10):

*“Overall, G1_T1map_ demonstrated consistent and significant correlations across both group- and subject-level (see* ***Supplementary Fig. 6*** *for the preliminary short-range structural connectivity analysis), while G1_MTsat_ results showed weaker and less consistent correlation.”*

and the revised *Discussio*n, where we now also outline the need for additional follow-up work to interrogate the U-fiber system (P.14):

*“While this study focused on characterizing surface-based SWM properties, analyzing short-range structural connectivity indicated that the SWM properties retain their relationship with local connectivity patterns. Building on this, future research will incorporate tractography-based approaches to directly investigate the organization and functional role of short-range, U-fiber bundles in SWM connectivity.”*
