# Supplementary material for: Microstructural profiles of the human superficial white matter and their associations to cortical geometry and connectivity
Source: PLoS Biol. 2026 Jan 30;24(1):e3003629. doi: 10.1371/journal.pbio.3003629 (PMC12885375; doi:10.1371/journal.pbio.3003629)
Supplement: Attachment — Submitted filename: response_SWM_PLOSBiology_final_auresp_3.docx [file pbio.3003629.s011.docx]

**Response to Editors and Reviewers (PBIOLOGY-D-25-01584R2)**

We would like to thank the Editors and Reviewers for the thoughtful guidance for our work, and are happy the latest version is now satisfactory.

**Reviewer #1**

**Authors have addressed all my concerns. Thanks.**

**Reviewer #2**

**The revised version of the manuscript and the author responses addressed the points raised in the previous round of review satisfactorily.**

**Reviewer #3**

**I have no further question and would recommend the acceptance of this paper.**

We thank the Reviewers for all the help and thoughtful suggestions for the paper.

**Editors**

****Title:**

**-- We see that you have already modified your study's title in the latest revision. However, we have another suggestion. We think that emphasizing the potential for biological insights from your paper instead of the methodological aspects would be inviting to a broader set of readers. Is this alternative version acceptable to you?**

**"Linking microstructural features of the human superficial white matter architecture with cortical geometry and connectivity"**

We have addressed the changed the title to “Microstructural profiles of the human superficial white matter and their associations to cortical geometry and connectivity”. We hope this is a reasonable compromise between the suggested variant and our initial version, as it also emphasizes biological insights.

****Financial disclosure statement:**

**-- Please add links in the Financial Disclosure statement in the manuscript details.**

This is now added.

****Ethics:**

**-- We acknowledge that your study uses previously-collected data. But as this data is from human subjects, please include in your ethics statement information about consent.**

This is now added.

****Data:**

**-- As supplementary document files are not proofread, please upload your supplementary figures as independent files, and include the legends in the main text file.**

We uploaded the supplementary figures and inserted the legends in the main text file.

**-- Thank you for providing the data, code, and analysis notebooks. We also require the numerical data underlying the figures. Can you please include a supplemental data file, titled "S1 Data" and with the filename "S1_Data.xlsx" including the numerical data? You can upload this file in the supplement or include it in an online deposition.**

**This applies to the following figure panels:**

**4B**

**5AB (rightmost panels)**

**S4 (rightmost panels)**

**S5 (rightmost panels)**

**S6C (rightmost panels)**

We added this file.

**--- Please also cite the location of the data clearly in all relevant main and supplementary Figure legends, e.g. “The data underlying this Figure can be found in S1 Data” or “The data underlying this Figure can be found in**

****Code availability:**

**-- Thank you for providing the underlying code in GitHub. However, because Github depositions can be readily changed or deleted, please make a permanent DOI’d copy (e.g. in Zenodo) and provide this URL in the manuscript and Data Availability Statement.**
